# Soft Mappings Space

**DOI:** 10.1155/2014/307292

**Published:** 2014-09-14

**Authors:** Taha Yasin Ozturk, Sadi Bayramov

**Affiliations:** Department of Mathematics, Faculty of Science and Letters, Kafkas University, 36100 Kars, Turkey

## Abstract

Various soft topologies are being introduced on a given function space soft topological spaces. In this paper, soft compact-open topology is defined in functional spaces of soft topological spaces. Further, these functional spaces are studied and interrelations between various functional spaces with soft compact-open topology are established.

## 1. Introduction

Many practical problems in economics, engineering, environment, social science, medical science, and so forth cannot be dealt with by classical methods, because classical methods have inherent difficulties. The reason for these difficulties may be due to the inadequacy of the theories of parameterization tools. Molodtsov [1] initiated the concept of soft set theory as a new mathematical tool for dealing with uncertainties. Maji et al. [2, 3] research deals with operations over soft set. The algebraic structure of set theories dealing with uncertainties is an important problem. Many researchers heve contributed towards the algebraic structure of soft set theory. Aktaş and Çağman [4] defined soft groups and derived their basic properties. Acar et al. [5] introduced initial concepts of soft rings. Feng et al. [6] defined soft semirings and several related notions to establish a connection between soft sets and semirings. Shabir and Ali [7] studied soft ideals over a semigroup. Qiu-Mei et al. [8] defined soft modules and investigated their basic properties. Gunduz (Aras) and Bayramov [9, 10] introduced fuzzy soft modules and intuitionistic fuzzy soft modules and investigated some basic properties. Ozturk and Bayramov defined chain complexes of soft modules and their soft homology modules. Ozturk et al. introduced the concept of inverse and direct systems in the category of soft modules.

Recently, Shabir and Naz [11] initiated the study of soft topological spaces. Theoretical studies of soft topological spaces have also been by some authors in [12–17]. In the study [18] different soft point concepts from the studies [12–17] were given. In this study, soft point consepts in the study [18] are used.

In the present study, first soft compact set on soft topological spaces is defined and the properties of this soft compact set are investigated. Based on this, soft compact-open topology of soft topological spaces is defined on soft mapping space.

## 2. Preliminaries

In this section we will introduce necessary definitions and theorems for soft sets. Molodtsov [1] defined the soft set in the following way. Let *X* be an initial universe set and let *E* be a set of parameters. Let *P*(*X*) denote the power set of *X* and *A*, *B*⊆*E*.

**Definition 1 (see [1]).**

A pair (*F*, *A*) is called a soft set over *X*, where *F* is a mapping given by *F* : *A* → *P*(*X*).

In other words, the soft set is a parameterized family of subsets of the set *X*. For *e* ∈ *A*, *F*(*e*) may be considered as the set of *e*-elements of the soft set (*F*, *A*), or as the set of *e*-approximate elements of the soft set.

**Definition 2 (see [3]).**

For two soft sets (*F*, *A*) and (*G*, *B*) over *X*, (*F*, *A*) is called soft subset of (*G*, *B*) if

 (1) *A* ⊂ *B* and
 (2) for all *e* ∈ *A*, *F*(*e*) and *G*(*e*) are identical approximations.
 This relationship is denoted by $\left(F,A)\tilde{\subset }(G,B\right)$. Similarly, (*F*, *A*) is called a soft superset of (*G*, *B*) if (*G*, *B*) is a soft subset of (*F*, *A*). This relationship is denoted by $\left(F,A)\tilde{⊃}(G,B\right)$. Two soft sets (*F*, *A*) and (*G*, *B*) over *X* are called soft equal if (*F*, *A*) is a soft subset of (*G*, *B*) and (*G*, *B*) is a soft subset of (*F*, *A*).

**Definition 3 (see [3]).**

The intersection of two soft sets (*F*, *A*) and (*G*, *B*) over *X* is the soft set (*H*, *C*), where *C* = *A*∩*B* and for all *e* ∈ *C*, *H*(*e*) = *F*(*e*)∩*G*(*e*). This is denoted by $\left(F,A)\tilde{\cap }(G,B)=(H,C\right)$.

**Definition 4 (see [3]).**

The union of two soft sets (*F*, *A*) and (*G*, *B*) over *X* is the soft set, where *C* = *A* ∪ *B* and, for all *e* ∈ *C*,

(1) $$\begin{array}{c} H\left(ɛ\right)=\left\{\begin{array}{cc} F\left(e\right), & \text{if}\;\;e\in A-B,\\ G\left(e\right), & \text{if}\;\;e\in B-A,\\ F\left(e\right)\cup G\left(e\right) & \text{if}\;\;e\in A\cup B. \end{array}\right. \end{array}$$

This relationship is denoted by $\left(F,A)\tilde{\cup }(G,B)=(H,C\right)$.

**Definition 5 (see [3]).**

A soft set (*F*, *A*) over *X* is said to be a NULL soft set denoted by Φ if for all *e* ∈ *A*, *F*(*e*) = *⌀* (null set).

**Definition 6 (see [3]).**

A soft set (*F*, *A*) over *X* is said to be an absolute soft set denoted by $\tilde{X}$ if, for all *e* ∈ *A*, *F*(*e*) = *X*.

**Definition 7 (see [11]).**

The difference (*H*, *E*) of two soft sets (*F*, *E*) and (*G*, *E*) over *X*, denoted by (*F*, *E*)∖(*G*, *E*), is defined as *H*(*e*) = *F*(*e*)∖*G*(*e*) for all *e* ∈ *E*.

**Definition 8 (see [11]).**

Let *Y* be a nonempty subset of *X*, and then $\tilde{Y}$ denotes the soft set (*Y*, *E*) over *X* for which *Y*(*e*) = *Y*, for all *e* ∈ *E*.

In particular, (*X*, *E*) will be denoted by $\tilde{X}$.

**Definition 9 (see [11]).**

Let (*F*, *E*) be a soft set over *X* and *Y* be a nonempty subset of *X*. Then the subsoft set of (*F*, *E*) over *Y*, denoted by (^*Y*^ *F*, *E*), is defined as follows:  ^*Y*^ *F*(*e*) = *Y*∩*F*(*e*), for all *e* ∈ *E*. In other words $\left({}^{Y}F,E)=\tilde{Y}\cap (F,E\right)$.

**Definition 10 (see [19]).**

Let (*F*, *A*) and (*G*, *B*) be two soft sets over *X*_1_ and *X*_2_, respectively. The cartesian product (*F*, *A*)×(*G*, *B*) is defined by (*F* × *G*)_(*A*×*B*)_, where

(2) $$\begin{array}{c} {\left(F\times G\right)}_{\left(A\times B\right)}\left(e,k\right)=F\left(e\right)\times G\left(k\right),\;\;\;\forall \left(e,k\right)\in A\times B. \end{array}$$

According to this definiton, the soft set (*F*, *A*)×(*G*, *B*) is soft set over *X*_1_ × *X*_2_ and its parameter universe is *E*_1_ × *E*_2_.

**Definition 11 (see [11]).**

Let *τ* be the collection of soft set over *X*, and then *τ* is said to be a soft topology on *X* if

1. $\Phi ,\tilde{X}$ belongs to *τ*;
2. the union of any number of soft sets in *τ* belongs to *τ*;
3. the intersection of any two soft sets in *τ* belongs to *τ*.

The triplet (*X*, *τ*, *E*) is called a soft topological space over *X*.

**Definition 12 (see [11]).**

Let (*X*, *τ*, *E*) be a soft topological space over *X*, and then members of *τ* are said to be soft open sets in *X*.

**Definition 13 (see [11]).**

Let (*X*, *τ*, *E*) be a soft topological space over *X*. A soft set (*F*, *E*) over *X* is said to be a soft closed in *X*, if its relative complement (*F*, *E*)′ belongs to *τ*.

**Proposition 14 (see [11]).**

Let (*X*, *τ*, *E*) be a soft topological space over *X*. Then the collection *τ*_*e*_ = {*F*(*e*):(*F*, *E*) ∈ *τ*}, for each *e* ∈ *E*, defines a topology on *X*.

**Definition 15 (see [11]).**

Let (*X*, *τ*, *E*) be a soft topological space over *X* and let (*F*, *E*) be a soft set over *X*. Then the soft closure of (*F*, *E*), denoted by $\bar{\left(F,E\right)}$, is the intersection of all soft closed super sets of (*F*, *E*). Clearly $\bar{\left(F,E\right)}$ is the smallest soft closed set over *X* which contains (*F*, *E*).

**Definition 16 (see [18]).**

Let (*F*, *E*) be a soft set over *X*. The soft set (*F*, *E*) is called a soft point, denoted by (*x*_*e*_, *E*), if, for the element *e* ∈ *E*, *F*(*e*) = {*x*} and *F*(*e*′) = *⌀* for all *e*′ ∈ *E* − {*e*} (briefly denoted by *x*_*e*_).

**Definition 17 (see [18]).**

In the two soft points (*x*_*e*_, *E*) and (*y*_*e*′_, *E*) over a common universe *X*, we say that the points are different points if *x* ≠ *y* or *e* ≠ *e*′.

**Definition 18 (see [18]).**

Let (*X*, *τ*, *E*) be a soft topological space over *X*. A soft set (*F*, *E*) in (*X*, *τ*, *E*) is called a soft neighborhood of the soft point (*x*_*e*_, *E*)∈(*F*, *E*) if there exists a soft open set (*G*, *E*) such that (*x*_*e*_, *E*)∈(*G*, *E*)⊂(*F*, *E*).

**Definition 19 (see [20]).**

Let (*X*, *τ*, *E*) and (*Y*, *τ*′, *E*) be two soft topological spaces, and let *f* : (*X*, *τ*, *E*)→(*Y*, *τ*′, *E*) be a mapping. For each soft neighbourhood (*H*, *E*) of (*f*(*x*)_*e*_, *E*), if there exists a soft neighbourhood (*F*, *E*) of (*x*_*e*_, *E*) such that *f*((*F*, *E*))⊂(*H*, *E*), then *f* is said to be soft continuous mapping at (*x*_*e*_, *E*).

If *f* is soft continuous mapping for all (*x*_*e*_, *E*), then *f* is called soft continuous mapping.

**Definition 20 (see [20]).**

Let (*X*, *τ*, *E*) and (*Y*, *τ*′, *E*) be two soft topological spaces, and let *f* : *X* → *Y* be a mapping. If *f* is a bijection, soft continuous and *f* ^−1^ is a soft continuous mapping, then *f* is said to be soft homeomorphism from *X* to *Y*. When a homeomorphism *f* exists between *X* and *Y*, we say that *X* is soft homeomorphic to *Y*.

**Definition 21 (see [13]).**

Let (*X*, *τ*, *E*) be a soft topological space over *X*. A subcollection *β* of *τ* is said to be a base for *τ* if every member of *τ* can be expressed as a union of members of *β*.

**Definition 22 (see [13]).**

Let (*X*, *τ*, *E*) be a soft topological space over *X*. A subcollection *δ* of *τ* is said to be a subbase for *τ* if the family of all finite intersections members of *δ* forms a base for *τ*.

**Definition 23 (see [13]).**

Let {(*φ*_*s*_, *ψ*_*s*_):(*X*, *τ*, *E*)→(*Y*_*s*_, *τ*_*s*_, *E*_*s*_)}_*s*∈*S*_ be a family of soft mappings and {(*Y*_*s*_, *τ*_*s*_, *E*_*s*_)}_*s*∈*S*_ is a family of soft topological spaces. Then, the topology *τ* generated from the subbase *δ* = {(*φ*_*s*_, *ψ*_*s*_)_*s*∈*S*_^−1^(*F*, *E*):(*F*, *E*) ∈ *τ*_*s*_, *s* ∈ *S*} is called the soft topology (or initial soft topology) induced by the family of soft mappings {(*φ*_*s*_, *ψ*_*s*_)}_*s*∈*S*_.

**Definition 24 (see [13]).**

Let {(*X*_*s*_, *τ*_*s*_, *E*_*s*_)}_*s*∈*S*_ be a family of soft topological spaces. Then, the initial soft topology on *X* = ∏_*s*∈*S*_ *X*_*s*_ generated by the family {(*p*_*s*_, *q*_*s*_)}_*s*∈*S*_ is called product soft topology on *X*. (Here, (*p*_*s*_, *q*_*s*_) is the soft projection mapping from *X* to *X*_*s*_, *s* ∈ *S*.)

The product soft topology is denoted by ∏_*s*∈*S*_ *τ*_*s*_.

**Definition 25 (see [16]).**

Let (*X*, *τ*, *E*) be a soft topological space and *x*, *y* ∈ *X* such that *x* ≠ *y*. If there exist soft open sets (*F*, *A*) and (*G*, *B*) such that *x* ∈ (*F*, *A*), *y* ∈ (*G*, *B*), and (*F*, *A*)∩(*G*, *B*) = Φ, then (*X*, *τ*, *E*) is called a soft Hausdorff space.

**Definition 26 (see [13]).**

Let (*X*, *τ*, *E*) be a soft topological space. If there exists a soft finite subcovering of every soft open covering of the soft topological space (*X*, *τ*, *E*), then this soft space is called soft compact space.

**Definition 27 (see [18]).**

Let (*X*, *τ*, *E*) be a soft topological space over *X*. If, for every soft point (*x*_*e*_, *E*) in *X*, there exists a soft neighbothood (*G*, *E*) such that $\bar{\left(G,E\right)}$ is a soft compact subspace of *X*, then (*X*, *τ*, *E*) is said to be soft locally compact space.

## 3. Soft Mappings Space

Let {(*X*_*s*_, *τ*_*s*_, *E*)}_*s*∈*S*_ be a family of soft topological spaces over the same parameters set *E*. We define a family of soft sets (∏_*s*∈*S*_ *X*_*s*_, *τ*, *E*) as follows.

If *F*_*s*_ : *E* → *P*(*X*_*s*_) is a soft set over *X*_*s*_ for each *s* ∈ *S*, then ∏_*s*∈*S*_ *F*_*s*_ : *E* → *P*(∏_*s*∈*S*_ *X*_*s*_) is defined by (∏_*s*∈*S*_ *F*_*s*_)(*e*) = ∏_*s*∈*S*_ *F*_*s*_(*e*). Let us consider the topological product (∏_*s*∈*S*_ *X*_*s*_, ∏_*s*∈*S*_ *τ*_*s*_, ∏_*s*∈*S*_ *E*) of a family of soft topological spaces {(*X*_*s*_, *τ*_*s*_, *E*)}_*s*∈*S*_. We take the contraction to the diagonal Δ ⊂ ∏_*s*∈*S*_ *E* of each soft set *F* : ∏_*s*∈*S*_ *E* → *P*(∏_*s*∈*S*_ *X*_*s*_). Since there exists a bijection mapping between the diagonal Δ and the parameters set *E*, then the contractions of soft sets are soft sets over *E*.

Now, let us define the topology on (∏_*s*∈*S*_ *X*_*s*_, *E*). Let (*p*_*s*_0__, 1_*E*_):(∏_*s*∈*S*_ *X*_*s*_, *τ*, *E*)→(*X*_*s*_0__, *τ*_*s*_0__, *E*) be a projection mapping and let the soft set (*p*_*s*_0__, 1_*E*_)^−1^(*F*_*s*_0__, *E*) be for each (*F*_*s*_0__, *E*) ∈ *τ*_*s*_0__. Then

(3) ps0,1E−1Fs0,Eps0−1Fs0,E=Fs0×∏s≠s0X~s,E.

The soft topology is generated from $\{(F_{s_{0}}\times \prod_{s\neq s_{0}}{\tilde{X}}_{s},E)|s_{0}\in S$, (*F*_*s*_0__, *E*) ∈ *τ*_*s*_0__} as a soft subbase and this soft topology is denoted by *τ* = ∏_*s*∈*S*_ *τ*_*s*_.

**Definition 28 (see [21]).**

The soft topological space (∏_*s*∈*S*_ *X*_*s*_, *τ*, *E*) is called the product of family of the soft topological spaces {(*X*_*s*_, *τ*_*s*_, *E*)}_*s*∈*S*_.

Now, let the family of soft topological spaces {(*X*_*s*_, *τ*_*s*_, *E*)}_*s*∈*S*_ be disjoint; that is, *X*_*s*_1__∩*X*_*s*_2__ = *⌀* for each *s*_1_ ≠ *s*_2_. For the soft set *F* : *E* → ∪_*s*∈*S*_ *X*_*s*_ over the set *E*, define the soft set *F*|_*X*_*s*__ : *E* → *X*_*s*_ by

(4) $$\begin{array}{c} {\left.F\right|}_{X_{s}}\left(e\right)=F\left(e\right)\cap X_{s},\;\;\;\forall e\in E \end{array}$$

and the soft topology *τ* define by

(5) $$\begin{array}{c} \left(F,E\right)\in \tau ⟺\left({\left.F\right|}_{X_{s}},E\right)\in \tau_{s}. \end{array}$$

It is clear that *τ* is a soft topology.

**Definition 29 (see [21]).**

A soft topological space (∪_*s*∈*S*_ *X*_*s*_, *τ*, *E*) is called soft topological sum of the family of soft topological spaces {(*X*_*s*_, *τ*_*s*_, *E*)}_*s*∈*S*_ and denoted by ⊕_*s*∈*S*_(*X*_*s*_, *τ*_*s*_, *E*).

Let (*X*, *τ*, *E*) and (*Y*, *τ*′, *E*) be two soft topological spaces. *Y* ^*X*^ denoted the all soft continuous mappings from the soft topological space (*X*, *τ*, *E*) to the soft topological space (*Y*, *τ*′, *E*); that is,

(6) YX=f,1E:X,τ,E⟶Y,τ′,E ∣ f,1E-a soft continuous map.

If (*F*, *E*) and (*G*, *E*) are two soft sets over *X* and *Y*, respectively, then we define the soft set (*G* ^*F*^, *E*) over *Y* ^*X*^ as follows:

(7) GFe=f,1E:X,τ,E⟶Y,τ′,E ∣ fFe⊂Ge   for each e∈E.

Now, let *x*_*α*_ ∈ (*X*, *τ*, *E*) be an any soft point. We define the soft mapping *e*_*x*_*e*__ : (*Y* ^*X*^, *E*) → (*Y*, *τ*′, *E*) by *e*_*x*_*e*__(*f*) = *f*(*x*_*e*_) = (*f*(*x*))_*e*_. This mapping is called an evaluation map. For the soft set (*G*, *E*) over *Y*, *e*_*x*_*e*__^−1^(*G*, *E*) = (*G* ^*x*_*e*_^, *E*) is satisfied. The soft topology that is generated from the soft sets {(*G* ^*x*_*e*_^, *E*)∣(*G*, *E*) ∈ *τ*′} as a subbase is called pointwise soft topology and denoted by *τ*_*p*_.

**Definition 30 (see [21]).**

(*Y* ^*X*^, *τ*_*p*_, *E*) is called a pointwise soft function space (briefly PISFS).

Now, we construct relationships between some function spaces. Let {(*X*_*s*_, *τ*_*s*_, *E*)}_*s*∈*S*_ be a family of pairwise disjoint soft topological spaces, let (*Y*, *τ*′, *E*) be a soft topological spaces and ∏_*s*∈*S*_(*Y* ^*X*_*s*_^, *τ*_*s*_*p*__, *E*), and let ⊕_*s*∈*S*_(*X*_*s*_, *τ*_*s*_, *E*) be a product and sum of soft topological spaces, respectively. Define

(8) $$\begin{array}{c} \nabla :\underset{s\in S}{\prod }\left(Y^{X_{s}},\tau_{s_{p}},E\right)⟶\left(Y^{{⊕}_{s\in S}X_{s}},{\left(\tau^{\prime {⊕}_{s\in S}}\right)}_{p},E\right) \end{array}$$

such that, for all {(*f*_*s*_, 1_*E*_)} ∈ ∏_*s*∈*S*_ *Y* ^*X*_*s*_^ and for all *x*_*e*_ ∈ ⊕_*s*∈*S*_(*X*_*s*_, *τ*_*s*_, *E*), ∇_*s*∈*S*_({*f*_*s*_})(*x*_*e*_) = *f*_*s*_0__(*x*_*e*_) = (*f*_*s*_0__(*x*))_*e*_, where *x*_*e*_ belongs to unique (*X*_*s*_0__, *τ*_*s*_0__, *E*). We define the mapping

(9) $$\begin{array}{c} \nabla^{-1}:\left(Y^{{⊕}_{s\in S}X_{s}},{\left(\tau^{\prime {⊕}_{s\in S}}\right)}_{p},E\right)⟶\underset{s\in S}{\prod }\left(Y^{X_{s}},\tau_{s_{p}},E\right) \end{array}$$

by ∇^−1^(*f*) = {*f*∘*i*_*s*_ = *f*|_*X*_*s*__ : *X*_*s*_ → *Y*} ∈ ∏_*s*∈*S*_(*Y* ^*X*_*s*_^, *τ*_*s*_*p*__, *E*) for each *f* : ⊕_*s*∈*S*_ *X*_*s*_ → *Y*. It is clear that the mapping ∇^−1^ is an inverse of the mapping ∇ [21].

Let (*X*, *τ*, *E*) be a soft topological space, let {(*Y*_*s*_, *τ*_*s*_, *E*)}_*s*∈*S*_ be a family of soft topological spaces, and let {(*f*_*s*_, 1_*E*_):(*X*, *τ*, *E*)→(*Y*_*s*_, *τ*_*s*_, *E*)}_*s*∈*S*_ be a family of soft mappings. For each soft point *x*_*e*_ ∈ (*X*, *τ*, *E*), we define the soft map *f* = Δ_*s*∈*S*_ *f*_*s*_ : (*X*, *τ*, *E*)→(∏_*s*∈*S*_ *Y*_*s*_, *τ*, *E*) by *f*(*x*_*e*_) = {*f*_*s*_(*x*_*e*_)}_*s*∈*S*_ = {(*f*_*s*_(*x*))_*e*_}_*s*∈*S*_. If *f* : (*X*, *τ*, *E*)→(∏_*s*∈*S*_ *Y*_*s*_, *τ*, *E*) is any soft mapping, then *f* = Δ_*s*∈*S*_ *f*_*s*_ is satisfied for the family of soft mappings {*f*_*s*_ = *p*_*s*_∘*f* : (*X*, *τ*, *E*)→(*Y*_*s*_, *τ*_*s*_, *E*)}_*s*∈*S*_.

Now, let {(*Y*_*s*_, *τ*_*s*_′, *E*)}_*s*∈*S*_ be the family of soft topological spaces, and let (*X*, *τ*, *E*) be a soft topological space. We define mapping

(10) $$\begin{array}{c} \Delta :\underset{s\in S}{\prod }\left(Y^{X_{s}},\tau_{s_{p}}^{\prime },E\right)⟶\left({\left(\underset{s\in S}{\prod }Y_{s}\right)}^{X},{\left(\underset{s\in S}{\prod }\tau_{s}^{\prime }\right)}_{p},E\right) \end{array}$$

by the rule for all {*f*_*s*_ : *X* → *Y*_*s*_} ∈ ∏_*s*∈*S*_(*Y* ^*X*_*s*_^, *τ*_*s*_*p*__′, *E*), Δ{*f*_*s*_} = Δ_*s*∈*S*_ *f*_*s*_.

Let the mapping Δ^−1^ = ((∏_*s*∈*S*_ *Y*_*s*_)^*X*^, (∏_*s*∈*S*_ *τ*_*s*_′)_*p*_, *E*) → ∏_*s*∈*S*_(*Y* ^*X*_*s*_^, *τ*_*s*_*p*__′, *E*) be

(11) $$\begin{array}{c} \Delta^{-1}\left(f\right)=\left\{p_{s}\circ f=f_{s}:X_{s}⟶Y\right\} \end{array}$$

for each *f* ∈ ((∏_*s*∈*S*_ *Y*_*s*_)^*X*^, (∏_*s*∈*S*_ *τ*_*s*_′)_*p*_, *E*), where *p*_*s*_ : (∏_*s*∈*S*_ *Y*_*s*_, *τ*, *E*)→(*Y*_*s*_, *τ*_*s*_, *E*) is a soft projection mapping. It is clear that the mapping Δ^−1^ is an inverse of the mapping Δ [21].

Now, let (*X*, *τ*, *E*), (*Y*, *τ*′, *E*), and (*Z*, *τ*′′, *E*) be soft topological spaces and let *f* : (*Z*, *τ*′′, *E*) × (*X*, *τ*, *E*) → (*Y*, *τ*′, *E*) be a soft mapping. Then the induced map $\hat{f}:X\to Y^{Z}$ is defined by $\hat{f}(x_{\alpha })(z_{\beta })=f(x_{\alpha },z_{\beta })$ for soft points *x*_*α*_ ∈ (*X*, *τ*, *E*) and *z*_*β*_ ∈ (*Z*, *τ*′′, *E*). We define exponential law

(12) $$\begin{array}{c} E:Y^{Z\times X}⟶{\left(Y^{Z}\right)}^{X} \end{array}$$

by using induced maps $E(f)=\hat{f}$; that is, $E(f)(x_{\alpha })\left(z_{\beta }\right)=f(z_{\beta },x_{\alpha })=\hat{f}(x_{\alpha })(z_{\beta })$. We define the following mapping:

(13) $$\begin{array}{c} E^{-1}:{\left(Y^{Z}\right)}^{X}⟶Y^{Z\times X} \end{array}$$

which is an inverse mapping *E* as follows [21]:

(14) E−1f^f,E−1f^zβ,xαE−1f^xαzβ=fzβ,xα.

Let (*X*, *τ*, *E*) be a soft topological space and let (*H*, *E*) be a soft set. For *τ*_*H*_ = {(*F*, *E*)∩(*H*, *E*):(*F*, *E*) ∈ *τ*}, since the conditions

1. ∪_*α*_((*F*_*α*_, *E*)∩(*H*, *E*)) = ∪_*α*_((*F*_*α*_, *E*))∩(*H*, *E*) ∈ *τ*_*H*_,
2. ((*F*_1_, *E*)∩(*H*, *E*))∩((*F*_2_, *E*)∩(*H*, *E*)) = ((*F*_1_, *E*)∩(*F*_2_, *E*))∩(*H*, *E*) ∈ *τ*_*H*_

are satisfied, then (*H*, *τ*_*H*_, *E*) is soft topological space over (*H*, *E*).

**Definition 31 .**

The soft set (*H*, *E*) is called a soft compact set if the soft topological space (*H*, *τ*_*H*_, *E*) is a soft compact topological space.

**Theorem 32 .**

Let (*X*, *τ*, *E*) be a soft topological space. Then (*H*, *τ*_*H*_, *E*) is a soft compact topological space if and only if there is a soft finite subcovering of every soft open covering of the soft set (*H*, *E*) in the soft topological space (*X*, *τ*, *E*).

**Proof**

⇒ Suppose that (*H*, *τ*_*H*_, *E*) is soft compact topological set and {(*F*_*α*_, *E*)}_*α*∈*A*_ is a soft open covering of the soft set (*H*, *E*) in the soft topological space (*X*, *τ*, *E*). Then

(15) H,E⊂⋃α∈AFα,E   ⟹H,E=⋃α∈AFα,E∩H,E.

For ever each *α* ∈ *A*, since (*F*_*α*_, *E*)∩(*H*, *E*) ∈ *τ*_*H*_, then the family of soft sets {(*F*_*α*_, *E*)∩(*H*, *E*)}_*α*∈*A*_ is a soft open covering of the soft topological space (*H*, *τ*_*H*_, *E*) and since (*H*, *τ*_*H*_, *E*) is a soft compact, then

(16) ∃ α1,α2,…,αn:H,E=⋃i=1nFαi,E∩H,E=⋃i=1nFαi,E.

That is, the family ${\left\{(F_{\alpha_{i}},E)\right\}}_{i=\bar{1,n}}$ is a soft finite covering of the soft set (*H*, *E*).

⇐ Conversely, suppose the given condition holds. Let the family {(*G*_*α*_, *E*)}_*α*∈*A*_ be a soft open covering of the soft topological space (*H*, *τ*_*H*_, *E*). For each *α* ∈ *A*, since (*G*_*α*_, *E*) ∈ *τ*_*H*_, choose a soft set (*F*_*α*_*i*__, *E*) ∈ *τ* such that (*G*_*α*_, *E*) = (*H*, *E*)∩(*F*_*α*_*i*__, *E*). Then the family {(*F*_*α*_, *E*)}_*α*∈*A*_ is a soft open covering of (*H*, *E*) in the soft topological spaces (*X*, *τ*, *E*). From the hypothesis,

(17) ∃Fα1,E,…,Fαn,E: H,E⊂⋃i=1nFαi,E⟹H,E=H,E∩⋃i=1nFαi,E=⋃i=1nFαi,E∩H,E=⋃i=1nGαi,E.

That is, the soft topological space (*H*, *τ*_*H*_, *E*) is a soft compact.

**Theorem 33 .**

Every soft closed subset of a soft compact topological space is soft compact.

**Proof**

Let (*X*, *τ*, *E*) be a soft compact space, let (*H*, *E*) be a soft closed set, and let the family *ϑ*_*E*_ = {(*F*_*α*_, *E*)}_*α*∈*A*_ be a soft open covering of (*H*, *E*) in the soft topological spaces (*X*, *τ*, *E*). Then the family of sets *ϑ*_*E*_ ∪ (*H*, *E*)′ is a soft open covering of (*X*, *τ*, *E*). Since (*X*, *τ*, *E*) is a soft compact space, then

(18) ∃Fα1,E,…,Fαn,E: ⋃i=1nFαi,E∪H,E′=X,τ,E.

From here,

(19) $$\begin{array}{c} \left(H,E\right)\subset \overset{n}{\underset{i=1}{⋃}}\left(F_{\alpha_{i}},E\right). \end{array}$$

Hence from Theorem 32, (*H*, *E*) is soft compact set.

**Theorem 34 .**

Every soft compact subset of a soft Husdorff topological space is soft closed.

**Proof**

Let (*X*, *τ*, *E*) be a soft Hausdorff space and let (*H*, *E*) be a soft compact set. We will prove that (*H*, *E*)′ is soft open, so that (*H*, *E*) is soft closed. Each soft point *x*_*e*_ ∈ (*H*, *E*) to any the soft point *y*_*e*′_ ∈ (*H*, *E*)′ is not equal. Since (*X*, *τ*, *E*) is a soft Hausdorff space, for *x*_*e*_ ≠ *y*_*e*′_ ∈ (*X*, *E*)

(20) ∃Uxe,E,Vye′,Eτ:xe∈Uxe,E,ye′Vye′,E, Uxe,E∩Vye′,E=Φ~.

Then the family {(*V*_*y*_*e*′__, *E*)}_*y*_*e*′_∈(*H*,*E*)_ is a soft open covering of (*H*, *E*) in the soft topological spaces (*X*, *τ*, *E*). Since the soft set (*H*, *E*) is a soft compact set, then

(21) $$\begin{array}{c} \exists \left(V_{y_{e_{1}^{\prime }}^{1}},E\right),\ldots ,\left(V_{y_{e_{n}^{\prime }}^{n}},E\right):\left(H,E\right)\subset \overset{n}{\underset{i=1}{⋃}}\left(V_{y_{e_{i}^{\prime }}^{i}},E\right) \end{array}$$

can be written. We take the soft neighborhoods ${\left\{(U_{x_{e_{i}}^{i}},E)\right\}}_{i=\bar{1,n}}$ of the soft point *x*_*e*_ to the corresponding the soft sets $(V_{y_{e_{i}^{\prime }}^{i}},E),\;\;i=\bar{1,n}$ such that $(U_{x_{e_{i}}^{i}},E)\cap (V_{y_{e_{i}^{\prime }}^{i}},E)=\tilde{\Phi }$. The soft set (*U*_*x*_*e*__, *E*) = ⋂_*i*=1_^*n*^(*U*_*x*_*e*_*i*__^*i*^_, *E*) is a soft open neighborhood of the soft point *x*_*e*_ and

(22) H,E∩Uxe,E=H,E∩⋂i=1nUxeii,E ⊂⋃i=1nVyei′i,E∩⋂i=1nUxeii,E=⋃i=1nVyei′i,E∩⋂i=1nUxeii,E ⊂⋃i=1nVyei′i,E∩Uxeii,E=Φ~.

Hence *x*_*e*_ ∈ (*U*_*x*_*e*__, *E*)⊂(*H*, *E*)′ holds. That is, (*H*, *E*)′ is soft open set and (*H*, *E*) is soft closed set.

**Remark 35 .**

The union of finite number of soft compact set on soft topological spaces is soft compact set.

**Lemma 36 .**

If (*X*, *τ*, *E*) is a soft locally compact Hausdorff space, (*K*, *E*) is a soft compact set, and (*K*, *E*)⊂(*V*, *E*), (*V*, *E*) ∈ *τ*, and then there exists a soft open set (*U*, *E*) ∈ *τ* such that $(K,E)\subset (U,E)\subset \bar{\left(U,E\right)}\subset (V,E),\bar{\left(U,E\right)}$ is a soft compact set.

**Proof**

Since (*X*, *τ*, *E*) is a soft Hausdorff space, for each the soft point *x*_*e*_ ∈ (*K*, *E*)⊂(*V*, *E*) there exists a soft neighborhood (*V*_*x*_*e*__, *E*) such that $x_{e}\in (V_{x_{e}},E)\subset \bar{\left(V_{x_{e}},E\right)}\subset (V,E)$. Since (*X*, *τ*, *E*) is a soft locally compact space, for each the soft point *x*_*e*_ ∈ (*K*, *E*) there exists a soft neighborhood (*W*_*x*_*e*__, *E*) such that $\bar{\left(W_{x_{e}},E\right)}$ is soft compact. Hence, for soft neighborhood (*U*_*x*_*e*__, *E*) = (*V*_*x*_*e*__, *E*)∩(*W*_*x*_*e*__, *E*) of the soft point *x*_*e*_, since $\bar{\left(U_{x_{e}},E\right)}\subset \bar{\left(W_{x_{e}},E\right)}$, then $\bar{\left(U_{x_{e}},E\right)}$ is a soft compact. Since the soft set (*K*, *E*) is a soft compact, there exists the soft finite subcovering {(*U*_*x*_*e*_1__^1^_, *E*),…, (*U*_*x*_*e*_*n*__^*n*^_, *E*)} of the soft covering {(*U*_*x*_*e*__, *E*)}_*x*_*e*_∈(*K*,*E*)_ of (*K*, *E*). From here, for the soft open set (*U*, *E*) = ⋃_*i*=1_^*n*^(*U*_*x*_*e*_*i*__^*i*^_, *E*), the conditions

(23) K,EU,E,U,E¯⋃i=1nUxeii,E¯⊂⋃i=1nVxeii,E¯⊂V,E

are satisfied.

Let (*X*, *τ*, *E*) and (*Y*, *τ*′, *E*) be two soft topological spaces and let (*F*, *E*) and (*G*, *E*) be two soft sets over *X* and *Y*, respectively. *M*((*F*, *E*), (*G*, *E*)) ⊂ *Y* ^*X*^ is denoted a soft mappings set, where the condition (*f*, 1_*E*_)(*F*, *E*)⊂(*G*, *E*) is satisfied.

**Definition 37 .**

Let (*X*, *τ*, *E*) and (*Y*, *τ*′, *E*) be two soft topological spaces. The generated topology from the sets

(24) $$\begin{array}{c} \left\{M\left(\left(C,E\right),\left(U,E\right)\right)\;|\;\left(C,E\right)\text{-soft}\;\text{compact}\;\text{set},\;\left(U,E\right)\in \tau^{\prime }\right\} \end{array}$$

as a soft subbase is called soft compact-open topology. The soft set *M*((*C*, *E*), (*U*, *E*)) is briefly denoted by *M*_*C*,*U*_.

**Example 38 .**

Let *X* = {*x*_1_, *x*_2_}, *Y* = {*y*_1_, *y*_2_, *y*_3_} and *E* = {*e*_1_, *e*_2_}. If we give the soft sets *F*_*i*_ : *E* → *P*(*X*) and *G*_*j*_ : *E* → *P*(*Y*) for *i*, *j* ∈ *I* defined by

(25) F1e1=x1,F1e2=⊘,F2e1=⊘,F2e2=x2,F3e1=x1,F3e2=x2,Ge1=y1,y2,Ge2=y3,

then the families $\tau =\{\Phi ,\tilde{X},(F_{1},E),(F_{2},E),(F_{3},E)\}$ and $\tau^{\prime }=\{\Phi ,\tilde{Y},(G,E)\}$ are soft topology.

Now, let us give the soft continuous mappings set *Y* ^*X*^. (*f*_*i*_, 1_*E*_) : (*X*, *τ*, *E*)→(*Y*, *τ*′, *E*) consists of the mappings

(26) f1x1=y1,f1x2=y1,f2x1=y2,f2x2=y2,f3x1=y3,f3x2=y3,f4x1=y1,f4x2=y3,f5x1=y2,f5x2=y3.

Since the soft topological space is a finite, in this space each soft set is a soft compact. To give subbase of soft compact-open topology in the space *Y* ^*X*^, the function family {*f*_*i*_}_*i*∈*I*_ in which each soft set in the space (*X*, *τ*, *E*) moves to soft set (*G*, *E*) in (*Y*, *τ*′, *E*) should be generated.

If we take the soft set {*H*_1_(*e*_1_) = {*x*_1_}, *H*_1_(*e*_2_) = ⊘}, then

(27) $$\begin{array}{c} M\left(\left(H_{1},E\right),\left(G,E\right)\right)=\left\{f_{1},f_{2},f_{4},f_{5}\right\}. \end{array}$$

If we take the soft set {*H*_2_(*e*_1_) = {*x*_1_, *x*_2_}, *H*_2_(*e*_2_) = {*x*_2_}}, then

(28) $$\begin{array}{c} M\left(\left(H_{2},E\right),\left(G,E\right)\right)=⊘ \end{array}$$

and so forth.

**Theorem 39 .**

Let (*X*, *τ*, *E*) be soft locally compact Hausdorff space; let *Y* ^*X*^ have the soft compact open topology. Then the soft evaluation map

(29) $$\begin{array}{c} e:Y^{X}\times X⟶Y \end{array}$$

is soft continuous.

**Proof**

Given a soft point (*f*, *x*_*e*_) of *Y* ^*X*^ × *X* and a soft open set (*V*, *E*) in *Y* about the image soft point *e*(*f*, *x*_*e*_) = *f*(*x*_*e*_), we wish to find a soft open about (*f*, *x*_*e*_) that *e* maps into (*V*, *E*). First, using the soft continuity (*f*, 1_*E*_) and the fact that (*X*, *τ*, *E*) is soft locally compact Hausdorff space, we can choose a soft open set (*U*, *E*) about *x*_*e*_ having soft compact closure $\bar{\left(U,E\right)}$, such that (*f*, 1_*E*_) carries $\bar{\left(U,E\right)}$ into (*V*, *E*). Then consider the soft open set $V^{\bar{U}}\times U$ in *Y* ^*X*^ × *X*. It is a soft open set containing (*f*, *x*_*e*_) and $e(V^{\bar{U}}\times U)\subset (V,E)$.

**Lemma 40 .**

Let the soft set (*K*, *E*) be a soft compact set in the soft topological space (*X*, *τ*, *E*) and let *y*_*e*′_ be a soft point in the soft topological space (*Y*, *τ*′, *E*). Let (*W*, *E*) be an arbitrary soft open set in the soft product space *X* × *Y* containing the soft set (*K*, *E*) × *y*_*e*′_. Then the soft opens (*U*, *E*) ∈ *τ* and (*V*, *E*) ∈ *τ*′ can be found such that

(30) $$\begin{array}{c} \left(K,E\right)\times y_{e^{\prime }}\subset \left(U\times V,E\right)\subset \left(W,E\right). \end{array}$$

**Proof**

For each the soft point *x*_*e*_ ∈ (*K*, *E*), (*x*_*e*_, *y*_*e*′_)∈(*W*, *E*) is satisfied. From the definition of soft topological product space, there exist the soft sets (*U*_*x*_*e*__, *E*) ∈ *τ* and (*V*_*y*_*e*′__, *E*) ∈ *τ*′ such that

(31) $$\begin{array}{c} \left(x_{e},y_{e^{\prime }}\right)\in \left(U_{x_{e}}\times V_{y_{e^{\prime }}},E\right)\subset \left(W,E\right). \end{array}$$

Then the soft family {(*U*_*x*_*e*__ × *V*_*y*_*e*′__, *E*)}_*x*_*e*_∈(*K*,*E*)_ is a soft open covering of the soft set (*K*, *E*) × *y*_*e*′_. Since (*K*, *E*) is a soft compact, the soft set (*K*, *E*) × *y*_*e*′_ is also soft compact. Then

(32) ∃ xe11,…,xenn∈K,E: K,E×ye′⊂⋃i=1nUxeii×Vyei′i,E

is written. From here, the soft open sets (*U*, *E*) = ⋃_*i*=1_^*n*^(*U*_*x*_*e*_*i*__^*i*^_, *E*) and (*V*, *E*) = ⋂_*i*=1_^*n*^(*V*_*y*_*e*_*i*_′_^*i*^_, *E*) are satisfied in the condition of lemma.

**Theorem 41 .**

Let (*Z*, *τ*′′, *E*) be a soft locally compact Hausdorff space, and let (*X*, *τ*, *E*) and (*Y*, *τ*′, *E*) be arbitrary two soft topological spaces. Let *Y* ^*Z*^ have the soft compact open topology. Then a map *f* : *Z* × *X* → *Y* is soft continuous if and only if $\hat{f}:X\to Y^{Z}$ is soft continuous.

**Proof**

Suppose that $\hat{f}$ is a soft continuous. Consider the functions

(33) $$\begin{array}{c} Z\times X\overset{1_{Z}\times \hat{f}}{\to }Z\times Y^{Z}\overset{t}{⟶}Y^{Z}\times Z\overset{e}{⟶}Y, \end{array}$$

where 1_*Z*_ denotes the soft identity function on *Z* and *t* denotes the switching map. Since

(34) e∘t∘1Z×f^ze′′,xe=e∘t∘ze′′,f^xe=f^xeze′′=fze′′,xe,

it follows that.

$f=e\circ t\circ (1_{Z}\times \hat{f})$, and *f* being the composition of soft continuous maps is itself soft continuous map.

Conversly suppose that *f* is soft continuous map. We prove that $\hat{f}$ is soft continuous. For any soft point *x*_*e*_ in *X*, let soft set *M*_*K*,*V*_ which belongs to subbase of (*Y* ^*Z*^, (*τ*′)^*τ*′′^, *E*) be an arbitrary neighborhood of soft point $\hat{f}(x_{e})$.

For proving the soft continuity of $\hat{f}$, we want to show that there exists soft open neighborhood (*W*, *E*) ∈ *τ* of soft point *x*_*e*_ such that

(35) $$\begin{array}{c} \left(\hat{f},1_{E}\right)\left(W,E\right)\subset M_{K,V}. \end{array}$$

Since $\hat{f}(x_{e})\left(z_{e^{\prime \prime }}\right)\subset (V,E)$, for each soft point *z*_*e*′′_ ∈ (*K*, *E*)⊂(*Z*, *E*),

(36) $$\begin{array}{c} \hat{f}\left(x_{e}\right)\left(z_{e^{\prime \prime }}\right)=f\left(z_{e^{\prime \prime }},x_{e}\right)\subset \left(V,E\right); \end{array}$$

thus (*f*, 1_*E*_)(*K* × *x*, *E*) ⊂ (*V*, *E*). Since *f* is soft continuous, (*f*, 1_*E*_)^−1^(*V*, *E*) is soft open set and

(37) $$\begin{array}{c} \left(K\times x,E\right)\subset {\left(f,1_{E}\right)}^{-1}\left(V,E\right). \end{array}$$

Hence by Lemma 40, there exists a soft open neighborhood (*W*, *E*) of *x*_*e*_ in (*X*, *τ*, *E*) such that

(38) $$\begin{array}{c} \left(K\times x,E\right)\subset \left(K\times W,E\right)\subset {\left(f,1_{E}\right)}^{-1}\left(V,E\right). \end{array}$$

Therefore (*f*, 1_*E*_)(*K* × *W*, *E*)⊂(*V*, *E*). Now, for any *u*_*e*_ ∈ (*W*, *E*) and *v*_*e*′_ ∈ (*K*, *E*),

(39) f^ueve′=fve′,ue⊂V,E;that is,  f^ue∈MK,V.

Hence $(\hat{f},1_{E})(W,E)\subset M_{K,V}$ as desired.

**Theorem 42 .**

Let (*X*, *τ*, *E*) and (*Z*, *τ*′′, *E*) be soft locally compact Housdorff spaces. Then for any soft topological spaces (*Y*, *τ*′, *E*), the function

(40) $$\begin{array}{c} E:Y^{Z\times X}⟶{\left(Y^{Z}\right)}^{X} \end{array}$$

is a soft homeomorphism.

**Proof**

Let us show that soft mapping

(41) $$\begin{array}{c} E:{\left(Y,\tau^{\prime },E\right)}^{\left(Z\times X,\tau^{\prime \prime }\times \tau ,E\right)}⟶{\left[{\left(Y,\tau^{\prime },E\right)}^{\left(Z,\tau^{\prime \prime },E\right)}\right]}^{\left(X,\tau ,E\right)} \end{array}$$

is a soft homeomorphism. The soft mapping *E* is bijective. Now, let us prove that soft mappings *E* and *E* ^−1^ are soft continuous. For proving the soft continuity of *E*, consider arbitrary soft open neighborhood *M*_*K*,*V*_ of soft point $E(f)=\hat{f}$ for any soft point *f* ∈ *Y* ^*Z*×*X*^, where *M*_*K*,*V*_ belongs to subbase. Here (*K*, *E*)⊂(*X*, *E*) is a soft compact and (*W*, *E*) ∈ *Y* ^*Z*^ is soft open in soft soft compact open topology. Without loss of generality we may assume that soft set (*W*, *E*) = *M*_*P*,*U*_, where (*P*, *E*)∈(*Z*, *E*) is soft compact and (*U*, *E*)∈(*Y*, *E*) is soft open set. Thus $\left(\hat{f},1_{E})(K,E)(P,E)\subset (U,E\right)$, and this implies that (*f*, 1_*E*_)(*P* × *K*, *E*) ⊂ (*U*, *E*). Since the soft sets (*P*, *E*) and (*K*, *E*) are soft compact, (*P* × *K*, *E*) is also soft compact. Then *M*_*P*×*K*,*U*_ is a soft open set in *Y* ^*Z*×*X*^. We conclude that *f* ∈ *M*_*P*×*K*,*U*_; that is, *M*_*P*×*K*,*U*_ is a soft open neighborhood of soft point *f*. Now we assert that *E*(*M*_*P*×*K*,*U*_) ⊂ *M*_*K*,*V*_. For any *g* ∈ *M*_*P*×*K*,*U*_, (*g*, 1_*E*_)(*P* × *K*, *E*) ⊂ (*U*, *E*).

Since $E(g)(x_{e})=\hat{g}(x_{e})$, $E(g)(x_{e})(z_{e^{\prime \prime }})=\hat{g}(x_{e})(z_{e^{\prime \prime }})=g(z_{e^{\prime \prime }},x_{e})\in (U,E)$ for each *x*_*e*_ ∈ (*K*, *E*) and *z*_*e*′′_ ∈ (*P*, *E*), *E*(*g*) ∈ *M*_*K*,*V*_ is satisfied.

Now for proving the soft continuity of *E* ^−1^, consider soft open neighborhood *M*_*Q*,*U*_ of $E^{-1}(\hat{f})=f\in Y^{Z\times X}$ for any soft point $\hat{f}\in {\left(Y^{Z}\right)}^{X}$. Since (*Q*, *E*)⊂(*Z* × *X*, *E*) is soft compact, (*Q*, *E*) = (*P* × *K*, *E*) is a product of soft compact sets. Since *f* ∈ *M*_*P*×*K*,*U*_, (*f*, 1_*E*_)(*P* × *K*, *E*) ⊂ (*U*, *E*). Let us consider soft open set *M*_*P*,*M*_*K*,*U*__ on soft space (*Y* ^*Z*^)^*X*^. For any soft points *x*_*e*_ ∈ (*P*, *E*), *z*_*e*′′_ ∈ (*K*, *E*), $\hat{f}(x_{e})(z_{e^{\prime \prime }})=f(z_{e^{\prime \prime }},x_{e})\in (U,E)$. Thus we have $\hat{f}\subset M_{P,M_{K,U}}$; that is, *M*_*P*,*M*_*K*,*U*__ is a soft open neighborhood of soft point $\hat{f}$.

Now we want to check that *E* ^−1^(*M*_*P*,*M*_*K*,*U*__) ⊂ *M*_*P*×*K*,*U*_. For any $\hat{g}\in M_{P,M_{K,U}}$ and arbitrary *x*_*e*_ ∈ (*P*, *E*), *z*_*e*′′_ ∈ (*K*, *E*),

(42) $$\begin{array}{c} E^{-1}\left(\hat{g}\right)\left(z_{e^{\prime \prime }},x_{e}\right)=g\left(z_{e^{\prime \prime }},x_{e}\right)\in \left(U,E\right); \end{array}$$

hence, *E* ^−1^ is soft continuous. Hence *E* is soft homeomorphism.

**Theorem 43 .**

If {(*X*_*s*_, *τ*_*s*_, *E*)}_*s*∈*S*_ is a family of soft topological spaces and (*Y*, *τ*′, *E*) is an arbitrary soft topological space, then the mapping

(43) $$\begin{array}{c} \nabla :\underset{s\in S}{\prod }\left(\left(Y^{X_{s}},{\left(\tau^{\prime }\right)}^{\tau_{s}},E\right)\right)⟶\left(Y^{{⊕}_{s\in S}X_{s}},{\left(\tau^{\prime }\right)}^{{⊕}_{s\in S}\tau_{s}},E\right) \end{array}$$

is a soft homeomerphism on soft compact open topology.

**Proof**

Consider an arbitrary soft open set *M*_*K*,*U*_ which belongs to subbasis of the soft space *Y* ^⊕_*s*∈*S*_*X*_*s*_^. Since (*K*, *E*) ⊂ (⊕_*s*∈*S*_ *X*_*s*_, *E*) is soft compact in soft topological sum, (*K*, *E*) = (*K*_*s*_1__, *E*)∪⋯∪(*K*_*s*_*n*__, *E*), (*K*_*s*_*i*__, *E*)⊂(*X*_*s*_*i*__, *E*)-soft compact. Then since

(44) ∇−1MK,U=MKs1,U×⋯×MKsn,U×∏s≠s1,…,snYXs,E

is soft open, ∇ is soft continuous.

If (*M*_*K*_*s*_1__,*U*_ × ⋯×*M*_*K*_*s*_*n*__,*U*_ × ∏_*s*≠*s*_1_,…,*s*_*n*__ *Y* ^*X*_*s*_^, *E*) which belongs to base in (∏*Y* ^*X*_*s*_^, *E*) is an arbitrary soft open set, then

(45) ∇MKs1,U×⋯×MKsn,U×∏s≠s1,…,snYXs,E =MKs1∪⋯∪Ksn,U1∪⋯∪Un

is soft open mapping. Since ∇ is injective, surjective, continuous, and soft open mapping, it is soft homeomorphism.

**Theorem 44 .**

Let (*X*, *τ*, *E*) be a soft topological space and let {(*Y*_*s*_, *τ*_*s*_′, *E*)}_*s*∈*S*_ be a family of soft topological spaces. The mapping

(46) Δ:∏s∈SYsX,τs′τ,E⟶∏s∈SYsX,∏s∈Sτs′τ,E

is a soft homeomorphism in soft compact open topology.

**Proof**

Let *M*_*K*,*U*_ be an arbitrary soft set which belongs to subbasis in soft function space (∏_*s*∈*S*_ *Y*_*s*_)^*X*^, where (*K*, *E*)⊂(*X*, *E*) is soft compact and (*U*, *E*) ⊂ (∏_*s*∈*S*_ *Y*_*s*_, *E*) is soft open set. We may take soft set (*U*, *E*) as

(47) $$\begin{array}{c} \left(U,E\right)=\left(U_{s_{1}}\times \cdots \times U_{s_{n}}\times \underset{s\neq s_{1},\ldots ,s_{n}}{\prod }Y_{s},E\right). \end{array}$$

Then since

(48) Δ−1MK,Us1×⋯×Usn=MK,Us1×⋯×MK,Usn×∏s≠s1,…,snYs

is soft open, Δ is soft continuous mapping.

For (*M*_*K*,*U*_*s*_1___ × ⋯×*M*_*K*,*U*_*s*_*n*___ × ∏_*s*≠*s*_1_,…,*s*_*n*__ *Y*_*s*_, *E*) which belongs to base is an arbitrary soft set, since

(49) ΔMK,Us1×⋯×MK,Usn×∏s≠s1,…,snYs,E =MKs1∪⋯∪Ksn,Us1×⋯×Usn×∏s≠s1,…,snYs

is soft open set, Δ is soft open mapping. This proves that Δ is a soft homeomorphism.

## 4. Conclusion

First soft compact set on soft topological spaces is defined and the properties of this soft compact set are investigated. Based on these concepts, soft compact-open topology is defined in functional spaces of soft topological spaces. Some properties of these functional spaces are studied. Proof of theorem of evaluation map, exponential law, and others are provided. Further, these functional spaces are studied and interrelations between various functional spaces soft compact-open topology are established. To extend this work, by using soft compact-open topology on function spaces, one could study different subdisciplines of mathematics, for example, algebraic topology, functional analysis, and so forth.
